# Functional analysis and cryo-electron microscopy of *Campylobacter* *jejuni* serine protease HtrA

**DOI:** 10.1080/19490976.2020.1810532

**Published:** 2020-09-22

**Authors:** Urszula Zarzecka, Alessandro Grinzato, Eaazhisai Kandiah, Dominik Cysewski, Paola Berto, Joanna Skorko-Glonek, Giuseppe Zanotti, Steffen Backert

**Affiliations:** a Division of Microbiology, Department of Biology, Friedrich-Alexander-University Erlangen-Nuremberg, Erlangen, Germany; b Department of General and Medical Biochemistry, Faculty of Biology, University of Gdańsk, Gdańsk, Poland; c Department of Biomedical Sciences, University of Padua, Padova, Italy; d European Synchrotron Radiation Facility (ESRF), Grenoble, France; e Mass Spectrometry Laboratory, Institute of Biochemistry and Biophysics, Polish Academy of Science, Warsaw, Poland

**Keywords:** *Campylobacter jejuni*, HtrA, protease, oligomerization, cryo-EM, cleavage site specificity, thermal stability

## Abstract

*Campylobacter jejuni* is a predominant zoonotic pathogen causing gastroenteritis and other diseases in humans. An important bacterial virulence factor is the secreted serine protease HtrA (HtrA*_Cj_*), which targets tight and adherens junctional proteins in the gut epithelium. Here we have investigated the function and structure of HtrA*_Cj_* using biochemical assays and cryo-electron microscopy. Mass spectrometry analysis identified differences and similarities in the cleavage site specificity for HtrA*_Cj_* by comparison to the HtrA counterparts from *Helicobacter pylori* and *Escherichia coli*. We defined the architecture of HtrA*_Cj_* at 5.8 Å resolution as a dodecamer, built of four trimers. The contacts between the trimers are quite loose, a fact that explains the flexibility and mobility of the dodecameric assembly. This flexibility has also been studied through molecular dynamics simulation, which revealed opening of the dodecamer to expose the proteolytically active site of the protease. Moreover, we examined the rearrangements at the level of oligomerization in the presence or absence of substrate using size exclusion chromatography, which revealed hexamers, dodecamers and larger oligomeric forms, as well as remarkable stability of higher oligomeric forms (> 12-mers) compared to previously tested homologs from other bacteria. Extremely dynamic decay of the higher oligomeric forms into lower forms was observed after full cleavage of the substrate by the proteolytically active variant of HtrA*_Cj_*. Together, this is the first report on the in-depth functional and structural analysis of HtrA*_Cj_*, which may allow the construction of therapeutically relevant HtrA*_Cj_* inhibitors in the near future.

## Introduction

*Campylobacter jejuni* is an important Gram-negative human pathogen responsible for gastrointestinal infections known as campylobacteriosis. The European Food Safety Authority (EFSA) and the European Center for Disease Prevention and Control (ECDC) reported in 2017 that campylobacteriosis had become the most commonly reported zoonosis in the European Union, representing almost 70% of all the reported cases of infection in humans.^1^ Although the infection is self-limiting in most cases, in a subset of individuals campylobacteriosis may lead to Guillain-Barré syndrome (GBS) or Miller Fisher syndrome, which are autoimmune conditions.^2^ In addition, a correlation was observed between various pathological gastrointestinal conditions such as inflammatory bowel diseases (IBD), Barrett’s esophagus, colorectal cancer and *C. jejuni* infection.^3^ The optimum temperature for growth of *C. jejuni* is 42°C, which means that the bacteria adapted to the body temperature in birds. Thus, *C. jejuni* can be frequently isolated from chicken and other poultry, which serve as hosts and reservoirs that are colonized asymptomatically.^4,5^ Moreover, *C. jejuni* and *Campylobacter coli* together are responsible for more than 95% of *Campylobacter* infections in humans.^6^ The potential sources of *C. jejuni* infections are handling or consumption of contaminated (undercooked) meat, cross-contaminated other foods, unpasteurized milk, contaminated water, or direct animal contact via household pets and farm animals.^7^

The molecular mechanisms responsible for *C. jejuni* virulence are not fully understood. Genomic analyzes have shown that they do not express classical bacterial virulence factors such as secretion systems or toxins that are often produced by other diarrhea-causing pathogens.^8^ The bacteria are highly motile, and this motility is an important aspect of virulence.^9^ Motility is required for colonization in mice, in a recently developed mouse model,^10^ while strains with impaired motility had a reduced ability to establish long-term colonization in chickens.^11^ Bacterial adherence to and entrance into intestinal epithelial cells are considered as critical steps for development of symptoms following *C. jejuni* exposure.^12,13^ *C. jejuni* expresses at least one dominant adhesin, called CadF, which was shown to bind fibronectin.^14,15^ CadF directly or indirectly promotes the binding of *C. jejuni* to polarized cells and may be an important virulence determinant.^16^ In addition, *C. jejuni* is capable of secreting certain proteins called *Campylobacter* invasion antigens (Cia proteins)^17^ whose functions are not fully known, but inactivation of at least CiaB resulted in a significant reduction in the number of *C. jejuni* internalized in host cells.^18^

All bacteria have effective stress responses to limit protein damage under harsh environmental conditions. In addition to the common cytoplasmic stress response proteins (DnaK, GroES/EL, GrpE, DnaJ, ClpB, etc.),^19,20^ the HtrA (high-temperature requirement A) protein plays an important protective function in the cellular envelope. This protein exhibits both protease and chaperone activities and is found in almost all bacteria.^21^ The best-characterized member of this protein family is HtrA from *Escherichia coli* (HtrA*_Ec_*, also known as DegP).^22,23^ As a protein quality control factor, DegP recognizes and degrades proteins that are not properly folded. In particular, DegP preferentially digests unfolded polypeptides with exposed hydrophobic residues and it mainly hydrolyzes peptide bonds after hydrophobic amino acid residues.^24^ A characteristic feature of the HtrA family of proteins is the presence of a chymotrypsin-type protease domain as well as one or two C-terminal PDZ domains (Postsynaptic density protein 95, *Drosophila* disc large tumor suppressor and Zonula occludens-1 protein domain).^25^ PDZ domains are typically involved in substrate binding, regulation of the proteolytic activity and inter-subunitinteractions.^26^

Over the past 20 years, 3D structures of several HtrA homologs have been determined. It has been found that all HtrAs form oligomers, whose building units are trimers. Generally, these proteases are present in two functional states, inactive (resting) and active, whose tertiary and quaternary structures differ.^26^ Rearrangements of the HtrA oligomers are associated with substrate binding and degradation. For example, *E. coli* DegP is converted from the resting state hexamer into proteolytically active 12- or 24-mer in the presence of substrate molecules.^27^ The large oligomers are cage-like assemblies with the inner chambers capable to accommodate substrate polypeptides. The active sites are accessible from the inside of the cage; hence, the assembly and disassembly of higher-order oligomers is believed to precisely regulate the protease activity.^26^ Assembly into the high order oligomers is transient and the protein returns to the resting state as soon as the substrate is depleted.^27^ Interestingly, despite the overall similarity of the monomer architecture among the HtrA homologs, their quaternary structures may markedly differ, especially in terms of the size of oligomers and way of their assembly. As HtrAs are required for virulence in several pathogenic bacterial species, learning the details of the spatial structure and regulation of activity of these proteins is of great importance.

While *E. coli* expresses three HtrA homologs (DegP, DegQ and DegS), only one *htrA* gene was identified in the genomes of *C. jejuni*.^28^ We have demonstrated that *C. jejuni* HtrA is a periplasmic protein, but it can also be secreted into the extracellular environment.^29–31^ Extracellular HtrA*_Cj_* is involved in virulence as it cleaves the adherens junction protein E-cadherin, an important factor required for maintaining an intact epithelial barrier.^32,33^ In addition, we have shown that HtrA*_Cj_* can digest the tight junction protein occludin.^34^ Apart from its protease activity, the chaperone activity of non-secreted HtrA*_Cj_* has been studied as well, and it was found that this enables growth of *C. jejuni* under stress such as high temperatures or elevated oxygen concentrations. However, a combination of these two stresses requires both the HtrA*_Cj_* proteolytic and chaperone activities for bacterial survival.^35,36^ This suggests that HtrA*_Cj_* is responsible for removal of misfolded proteins to prevent their accumulation to a toxic level within the bacterium.

Although the *in vivo* functions of HtrA*_Cj_* were studied to some extent,^33,35,37^ knowledge about structure and biochemical properties of the HtrA*_Cj_* molecule is still incomplete. The presence of putative HtrA*_Cj_* oligomers (trimers, hexamers and 12-mers) was demonstrated by electrophoresis under native conditions,^35^ but no further detailed analysis was performed. In the present study, we have enriched this knowledge with structural data using size exclusion chromatography and Cryo-EM. Using two model substrates and mass spectrometry we also performed a detailed analysis of HtrA*_Cj_* proteolytic activity requirements, which are crucial for proper functioning of the HtrA protein.

## Results

### Analysis of the HtrA_Cj_ proteolytic activity

**Figure 1.**
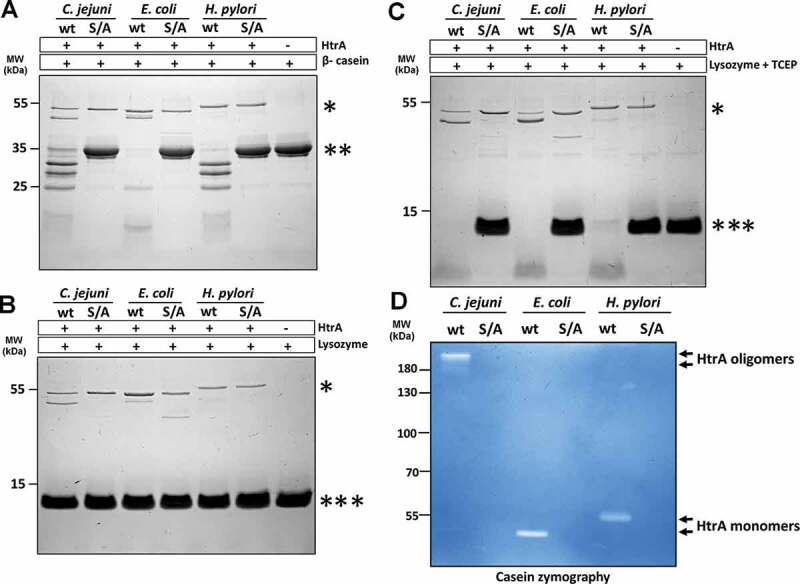
HtrA*_Cj_* is a serine protease that degrades unfolded or denatured proteins

To characterize the proteolytic activity of HtrA*_Cj_*, a folded and an unfolded protein substrate was incubated with the purified enzyme. For this purpose, we used β-casein, which is naturally unfolded^38^ and lysozyme,^39^ either in its native configuration or chemically reduced with TCEP (Figure 1). These substrates were incubated with recombinant active HtrA*_Cj_* or the proteolytically inactive S197A mutant. As expected, the latter protein had no effect on the substrates as control. Upon incubation with active HtrA*_Cj_*, however, degradation of β-casein and TCEP-treated lysozyme was complete after 90 min (Figure 1a,b). Similar results were obtained with HtrA/DegP from *E. coli* and HtrA from *H. pylori* (which is a closely related gastric pathogen, and contains a single HtrA homolog, similar to *C. jejuni*).^40^ Native lysozyme was resistant to HtrA*_Cj_* (Figure 1c). As an independent method to confirm the proteolytic activity of HtrA*_Cj_*, casein zymography was used, which again demonstrated digestion of this substrate (Figure 1d). Moreover, this method also allowed to observe that a certain fraction of HtrA*_Cj_* maintained its trimeric oligomeric form, despite the conditions of SDS-PAGE electrophoresis.

The proteolytically active HtrA (wt) and inactive variants S197A (S/A), of *C. jejuni, E. coli* and *H. pylori* were incubated with (a) β- casein or (b–c) lysozyme in 50 mM HEPES pH 7.4, 200 mM NaCl at 37°C for 90 min. The reactions with lysozyme were performed without (b) or with (c) 7 mM TCEP. As controls samples without HtrA were used. The molar ratio of HtrA/β-casein was 1:28; HtrA/lysozyme was 1:47. Proteins were resolved in 15% gels and stained with Coomassie Brilliant Blue. The picture shows representative examples of the experiments. The position of proteins was marked with asterisks: HtrA (*), β- casein (**) and lysozyme (***). (d) 50 ng of recombinant HtrA proteins (proteolytically active and inactive variants) were analyzed by casein zymography.

**Figure 2.**
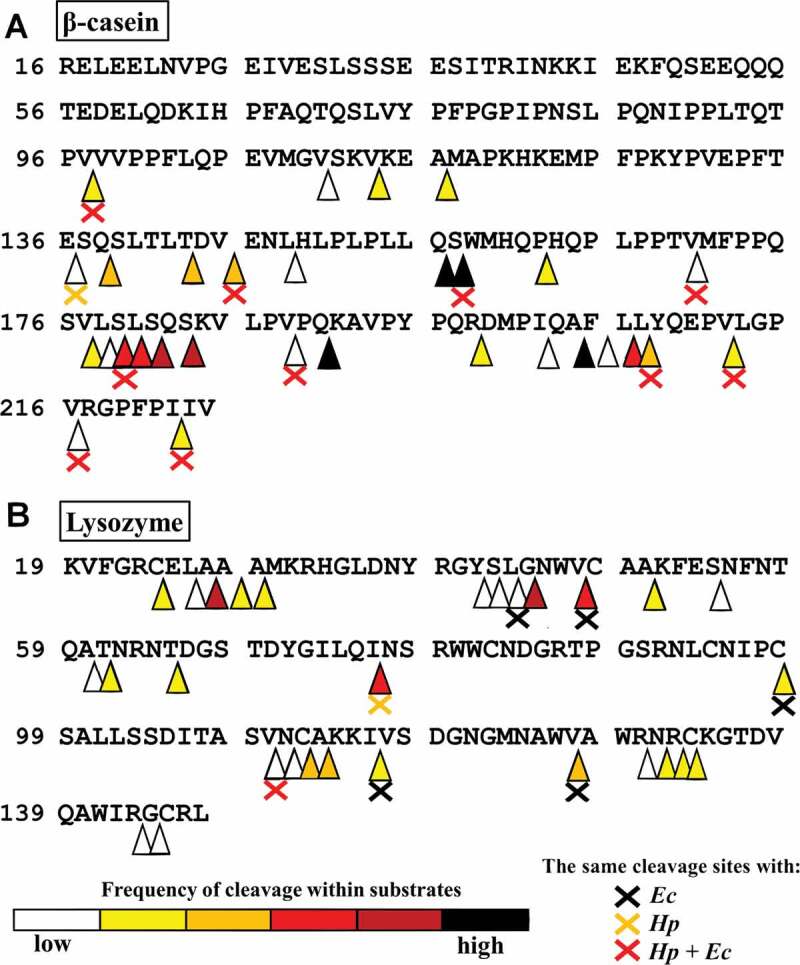
Position of HtrA*_Cj_* cleavage sites toward β-casein (a) and TCEP-treated lysozyme (b)

The model substrate digestion products were analyzed by mass spectrometry to identify sites preferentially cleaved by HtrA*_Cj_* (Figure 2 and Table S1). We found that the enzyme predominantly hydrolyzed the following peptide bonds in β-casein: Q_156_↓S_157_, S_157_↓W_158_, Q_190_↓K_191_ and A_204_↓F_205_, (black triangles), S↓Q, S↓K (dark-red triangles) and in reduced lysozyme: A_26_↓A_27_, G_44_↓N_45_, V_47_↓C_48_, and I_76_↓N_77_ (dark-red triangles in Figure 2). As shown, there is no defined preference for amino acid residues at the P1 position of the cleavage site. The S1 substrate specificity pocket obviously is able to accommodate both polar (Q, S) or nonpolar (A, V, I) residues. The same is true for the position P1ʹ of a cleaved bond: both large nonpolar (F, W) or polar/charged (S, N, K) residues can be found there. When the data were analyzed and normalized in respect of the relative frequency of the cleavage products and the relative abundance of each amino acid in the substrate protein, we found a slight preference for Q and S at position P1, but a strong preference of L, P and Q at position P2 (**Table S2**).

Digestion fragments were analyzed by mass spectrometry. The β-casein and lysozyme sequences are presented without signal sequence. The frequency of cleavage was determine based on signal intensity (at least 1 × 10^8^) and MS/MS count (at least 10) parameters **(Table S1)**. The start and end sites of the peptides were evaluated and the frequency of digestion in a given place is marked with heatmap-colored triangles. The crosses indicate similarity of published cleavage sites obtained with HtrA*_Hp_* and HtrA*_Ec_* (according to^41,42^).

### Thermal stability of the HtrA_Cj_ protein

It has been reported that HtrA*_Cj_* can reach its maximum proteolytic activity at 50–55°C.^35^ We considered the possibility that this observation relates to the thermal stability of the protein. For this purpose, we determined the melting curve at a temperature range of 20–95°C of the S197A mutant of HtrA*_Cj_* by means of circular dichroism (CD) and calculated the protein melting temperature (T_m_). The T_m_ value is regarded as a determinant of protein stability.^43^ As shown in Table 1, the melting point for HtrA*_Cj_* was approximately 78°C, and was higher than T_m_ of HtrA*_Ec_* and HtrA*_Sm_*, but lower by about 10°C compared to HtrA*_Hp_*.^41,42^ The T_m_ values seem to be in the agreement with the temperatures at which the enzymes exhibited their highest proteolytic activity (Table 1).

**Table 1.** Thermal stability of HtrA*_Cj_* S197A compared to other bacterial members of the HtrA family

	Tm [°C]	Reaction condition	Source	Maximum of proteolytic activity	Source
HtrA*_Cj_*	78.74 (±0.35)	pH 7.4	This publication	50–55°C (pH 8.0)	^35^
HtrA*_Ec_*	67.55–73.30	pH 5.5–8.0	^41,42^	55°C (pH 6.2)	^42^
HtrA*_Sm_*	56.11–61.34	pH 5.5–8.0	^41^	35–37°C (pH 6.2)	^41^
HtrA*_Hp_*	85.72–88.77	pH 5.5–8.0	^42^	75°C (pH 6.2)	^42^

### Cryo-EM of HtrA_Cj_ and structural description

**Figure 3.**
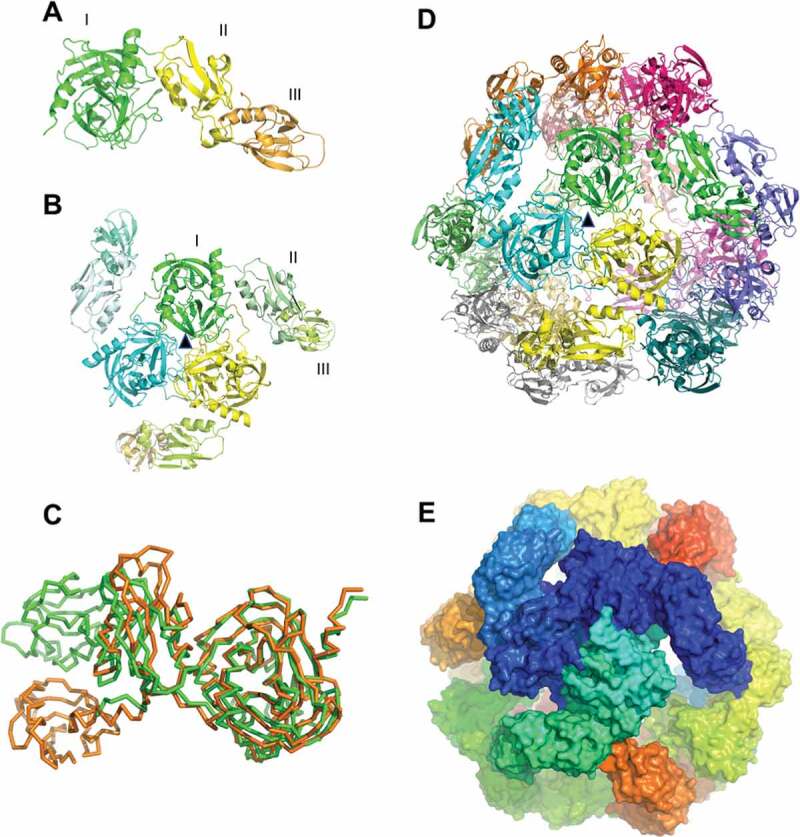
Three-dimensional modeling of *C. jejuni* HtrA (proteolytically inactive S197A mutant) using Cryo-electron microscopy

Since the proteolytically active HtrA*_Cj_* wild-type revealed a high tendency to auto-proteolysis, we expressed a proteolytically inactive recombinant S197A substitution mutant of HtrA*_Cj_* in *E. coli*, and after purification of the protein, the structure was investigated by Cryo-EM. Data analysis (see Methods) produced a 3D map at 5.8 Å resolution (**Table S3**). Although atomic details could not be obtained under the best conditions, this map allowed the characterization of the quaternary organization of the protein complex. HtrA*_Cj_* monomers have an elongated, bent shape with three well-separated structural domains (I–III): a central core domain that is responsible for the chymotrypsin-like protease (N-terminus up to residue 273) and two joining PDZ domains, PDZ1 (residues 275–362) and PDZ2 (residues 376–471) (Figure 3a). The relative positions of the three domains in the quaternary structure of the trimer and the organization of the dodecamer is observed with high confidence, but the actual conformation of the polypeptide chains, owing to the degree of resolution, could not be defined with certainty. In particular, the secondary structures within the proteolytic domain are reasonably well-defined, whilst the density of the other two domains, in particular PDZ2, is less defined.

(a) HtrA monomer showing the core domain (I, green), the first PDZ1 domain (II, yellow) and the second PDZ2 domain (III, orange). (b) Cartoon view of a trimer with the three monomers shown in green, yellow and cyan, and the three domains of each monomer represented in different shades of the same color. The three-fold axes that relates to the three monomers is shown as a black triangle, and runs roughly perpendicular to the represented plane. (c) Ribbon drawings showing the Cα atoms of monomeric HtrA*_Cj_* (green) superimposed on DegQ of *Legionella pneumophila* (orange, PDB ID 4YNN), demonstrating a different orientation of the PDZ2 domain. (d) Cartoon drawing and (E) surface representation of the dodecameric assembly of HtrA. Each monomer is shown with different color.

The basic unit of the structural organization of HtrA*_Cj_* is the trimer, with three monomers arranged around a three-fold axis. The three monomers interact via their proteolytic domains, whilst the two PDZ domains radiate outwards (Figure 3b). A comparison of the monomer of HtrA*_Cj_* with DegQ from *Legionella pneumophila*,^44^ which also contains two PDZ domains, shows that the protease and PDZ1 domains of the two proteins superpose quite well, while the most C-terminal PDZ2 domains are positioned at different angles in these two proteins (Figure 3c). A comparison of HtrA*_Cj_* with all the structures available at high resolution from *E. coli* of DegS (6ew9, 4rqz), DegP (3mh7, 1ky9), and DegQ (3sti, 3stj), shows that the catalytic domain present the same structure, and the same applies for the trimeric organization of the catalytic domains, when a trimer is present in the model. The situation of domains PDZ1 and PDZ2, however, is different. When present, the position of PDZ1 domain of HtrA*_Cj_* with respect to the catalytic one is similar to DegP (3mh7), DegS (6ew9, 1sot) and DegQ (3stj), but different to another model of DegP (1ky9) **(Figure S1)**. On the contrary, the PDZ2 domain presents an entirely different orientation to that of DegP (3mh7 and 1ky9). The structures of the plant HtrA homolog Deg9 (5il9) from *Arabidopsis* contain only the catalytic domain, whose conformation is similar to that of HtrA*_Cj_*. The structure of the different isoforms of the human protein is the same as far as the catalytic domain is concerned (HtrA1, 3num), albeit the orientation of PDZ1, when present, is different (HtrA3, 4ri0q; HtrA2, 1lcy).

**Figure 4.**
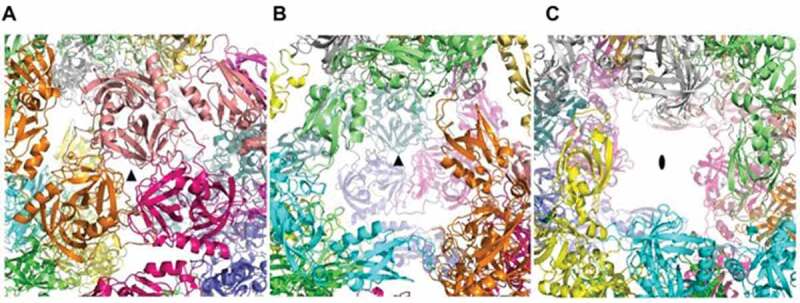
Three different types of openings are present in the HtrA dodecamer

In the higher-order assembly, four trimers of HtrA*_Cj_* arrange into a dodecamer, characterized by a tetrahedral symmetry (Figure 3d,e and Supplementary movie S1), consisting of 4 threefold rotation axes, running from each vertex of the tetrahedron to the center of an opposite face, and 3 twofold axes, running from the middle of each edge to the middle of an opposite edge. The contacts between trimers in the dodecamer are quite loose (Figure 3d), as the trimers are only held together by interactions between three PDZ2 domains of one trimer and the three PDZ1 domains of the other three trimers. This explains why the model is reasonably well defined in the central core of each trimer, but less defined for domain PDZ1 and rather approximate for domain PDZ2. The symmetry elements described above correspond to large or small openings of the dodecamer (Figure 4). Within each trimer, there is a small gap that may be permeable only to solvent molecules (Figure 4a), whilst the gap on the opposite side of the dodecameric assembly is quite large (Figure 4b), being a triangular aperture of about 36 Å from a vertex to an edge. Six large rectangular openings of about 32 Å x 24 Å are also present in correspondence of the 3 twofold axes (Figure 4c). These large gaps potentially allow the entrance of small or medium-sized molecules into the inner cavity. Both the external and internal surfaces of the dodecamer are strongly hydrophilic, with an almost uniform distribution of positive and negative charges (**Figure S2**).

The macromolecule has four such gaps as shown in (a), four as shown in (b) and six as shown in (c). (a) and (b) represents two different types of gaps that are related to a three-fold axis (indicated by the black triangle in the middle). (c) shows the gaps generated by the two-fold axes.

### Molecular dynamics of HtrA_Cj_

**Figure 5.**
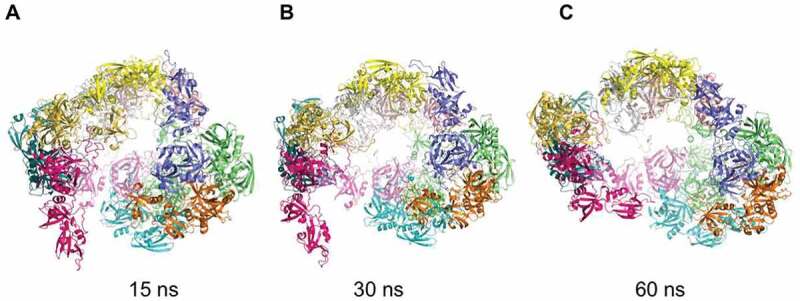
Structural dynamics of the dodecamer of *C. jejuni* HtrA

To investigate the stability of the dodecameric complex, a 100 ns molecular dynamics simulation was performed, starting from the cryo-EM structure. Already after 15 ns, the dodecamer opens up (Figure 5a) to result in a more open, oval configuration after 60 ns (Figure 5c). Nevertheless, the structure does not completely fall apart in trimers even after 100 ns, but fluctuates around this partially opened and flexible dodecameric assembly. The relative instability of the dodecamer is due to the very limited interactive surfaces between the four trimers, whilst the trimers themselves are quite stable.

Cartoon views are shown of a sequence of modeled HtrA structures after 15 ns (a), 30 ns (b) and 60 ns (c) of dynamics simulation. Already after 15 ns one trimer (whose monomers are colored magenta, dark blue and dark yellow) starts to separate, opening up the tetrameric structure and, generating a large gap in the quaternary structure. After 60 ns the entire dodecamer is affected and is reshaped into a more open, oval structure.

### HtrA_Cj_ oligomerization status

**Figure 6.**
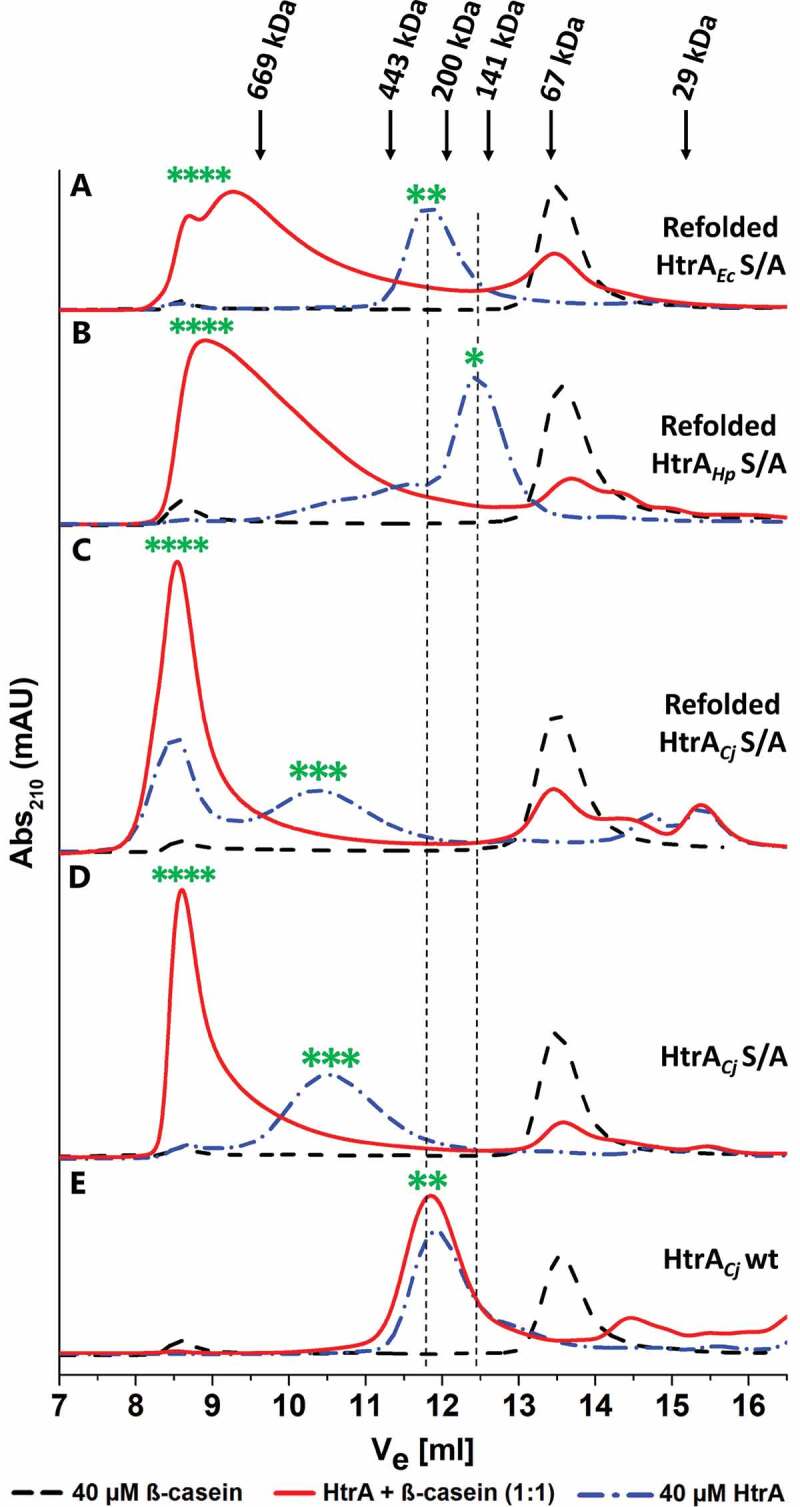
HtrA oligomerization in the presence of substrate

To further analyze the dynamics of the HtrA_Cj_ oligomers, we performed size exclusion chromatography (SEC) in the absence or in the presence of substrates (Figure 6). As controls, HtrA*_Ec_* and HtrA*_Hp_* proteins were included, whose oligomer states have been previously established.^41,42^ Proteolytically inactive S/A mutants of each of these proteins were purified in presence of urea and subsequently refolded to remove co-purifying peptides. As expected, in the absence of substrate, the refolded S/A mutant of HtrA*_Ec_* forms hexamers (Figure 6a) and that of HtrA*_Hp_* is mainly present as trimers with a small fraction of hexamers (Figure 6b), while large complexes are formed in presence of β-casein. In the absence of substrate, the refolded inactive S/A mutant of HtrA*_Cj_* was eluted at the position corresponding to dodecamer (Figure 6c), in accordance with the Cryo-EM data. The same results were obtained when the protein was purified under native conditions (Figure 6d). Incubation with substrate, β-casein, changed the elution profiles of all HtrA homologs and the large oligomeric forms were detected. In the case of HtrA*_Cj_*, the peak corresponding to large oligomers was sharper and more symmetric than these of HtrA*_Ec_* and HtrA*_Hp._* This suggests that the fraction of HtrA*_Cj_* oligomers was more homogenous, while the large oligomers of the control HtrAs are mixtures of oligomers of various sizes. It is also possible that the affinity of the control HtrAs to the substrate molecules is lower than that of HtrA*_Cj_* and the oligomers partially disassemble during the SEC experiment.

HtrA proteins purified with (a–c) or without (d, e) a refolding step were analyzed. Proteolytic inactive S/A mutants (a–d) and active wt (e) proteins were compared with HtrA*_Ec_* (a) and HtrA*_Hp_* (b) included as control). The proteins were analyzed without substrate (blue curves) and following pre-incubation with β-casein (molar ratio 1:1) (red curves), with substrate controls included (black curves). Arrows at the top indicate the elution volumes (V_e_) of molecular weight markers. The green asterisks indicate the putative position of trimers (*), hexamers (**), dodecamers (***) and larger oligomers (****). The dashed lines illustrate the position of trimers and hexamers.

We also noted that the elution profile of refolded HtrA*_Cj_* contained a peak corresponding to oligomers larger than dodecamers, suggesting presence of substrate peptides in the protein sample. This finding was surprising, as using denaturation and refolding step, we expected to obtain a preparation devoid of autocleaved peptides. However, examination of the refolded HtrA*_Cj_* samples revealed the presence of degradation products as evidenced by Western blot stained with anti-HtrA_Cj_ and anti-His tag antibodies, respectively **(Figure S3)**. Moreover, purification under denaturing conditions enriched the content of the large C-terminal HtrA*_Cj_* degradation product, containing His tag. We can expect that this peptide, due to its rather big size, was utilized by HtrA*_Cj_* as a substrate peptide and was responsible for formation of oligomers larger than 12-mers.

Interestingly, the molecules of proteolytically active HtrA*_Cj_* migrated as smaller particles, probably hexamers (Figure 6e) as judged from their elution volume (almost identical to that of the HtrA*_Ec_* hexamers).^41,42^ Pre-incubation with β-casein, however, did not change the elution pattern of the wild-type HtrA*_Cj_*, so we can expect that the active enzyme completely degraded its substrate and returned to the resting state.

## Discussion

Bacterial HtrA proteases constitute periplasmic enzymes involved in protein quality control, which is especially important under environmental stress conditions. When certain periplasmic proteins lose their conformation and functionality due to stressors, then HtrA acts as a protease and/or as a chaperone.^45^ In both cases, the level of toxic proteins is reduced. Another aspect of HtrA’s functioning is its role in the non-periplasmic space. In many pathogenic bacteria, including *H. pylori* and *C. jejuni*, the HtrAs can also target host cell factors.^21^ Such important functions, especially the proteolytic activity, must be strictly controlled, and in this case the quaternary organization can be involved. For this purpose, we focused our current research on: (i) proving that the HtrA*_Cj_* is able to digest unstructured β-casein and reduced lysozyme, while properly folded lysozyme remains undigested (Figure 1); (ii) determining that the HtrA*_Cj_* protein has high thermal stability which is in agreement with the ability of the protein to function under stress conditions^35^ (Table 1); (iii) demonstrating the preferred cleavage sites of this protease (Figure 2); (iv) establishing a 3D model of the dodecameric, structurally active form of HtrA*_Cj_* by Cryo-EM (Figure 3–5); and (v) performing analysis of the size of oligomeric forms for the proteolytically active HtrA*_Cj_* that allowed us to discover the basic, stable resting form for this protease-hexamer (Figure 6).

Experimental identification on protease cleavage sites suggests that HtrA*_Cj_* participates in the cellular protein quality control system of *C. jejuni*, as has been demonstrated for other HtrA homologs.^41,42,46,47^ Published data indicate that some HtrAs preferentially recognizes hydrophobic amino acid residues whose exposure is typical of improperly folded proteins.^22,48^ Interestingly, only one of the highly frequent cleavage sites (S_157_↓W_158_) was recognized by the HtrAs of *H. pylori* and *E. coli*. Cleavage sites less frequently recognized by HtrA*_Cj_* more often overlapped with those of the other two enzymes (Figure 2). Of all identified cleavage sites, 5 overlapped with those of HtrA*_Ec_* (all in lysozyme), two overlapped with those of HtrA*_Hp_* (one in each substrate) and 11 overlapped with both enzymes. This suggests some similarities in the recognized amino acid residues by these three studied HtrAs, as well as indicating that the sites preferred by HtrA_Cj_ may be slightly different for HtrA*_Hp_*, and HtrA*_Ec_*. These dfferences in substrate specificity have not resulted from sequence variations in the region of the S1 specificity pocket. In the case of HtrA*_Ec_*, it has been proven that this pocket is composed of I205 (the L1 loop), A227, and I239 (the L2 loop).^49^ Comparison of the amino acid sequences of HtrA*_Ec_* (DegP*_Ec_* and DegQ*_Ec_*), HtrA*_Cj_*, and HtrA*_Hp_* did not show variations in the S1 specificity pocket (**Figure S4**), suggesting the differences in substrate specificity are probably due to the conformational differences of HtrA molecules resulting in different accessibility of the active center.

The availability and selection of the substrates are crucial for the functioning of HtrAs. For example, HtrA*_Ec_* (DegP*_Ec_*) undergoes rearrangements between inactive and active conformations, which is allosterically (substrate-dependently) regulated and can be thermally induced.^50,51^ Improperly folded proteins or peptides act as allosteric activators and these bind simultaneously to the active center and the PDZ1 domain of the enzyme.^27,48^ Cryo-EM and SEC data shown in this paper help to understand the effect of substrates on HtrA*_Cj_* conformation. As we pointed out in the results, for HtrA*_Cj_* we encountered a problem, which was the presence of HtrA*_Cj_* degradation products in the purified protein samples, which contain histidine residues in their sequence (**Figure S3**) that hindered their removal. The binding of these peptides to HtrA*_Cj_* was also significant, because this prevents the separation of the smaller peptide by molecular filtration. Information about the presence of degradation fragments of HtrA*_Cj_* was published earlier.^35^ Our attempts to purify the protein under denaturing conditions and subsequent refolding was not successful to remove this fragment from the preparation, most probably due to the content of His-tag. As a consequence, the analyzed oligomerization profiles (using the SEC method) of this preparation showed the ability of HtrA*_Cj_* to create 12-mers and oligomers higher than 12-mers without additional substrate (Figure 6c). For testing, we also used a HtrA*_Cj_* variant purified under native conditions, which had less of the discussed degradation product. In this case, we observed the presence of the 12-mers (Figure 6d). Fragments co-purified with the protein are sufficient to initiate the formation of higher oligomeric forms, and the lack of proteolytic activity did not allow the substrate to be released. A similar problem was observed for DegQ from *Legionella*. A proteolytically inactive variant of protein was observed as a 12-mer, without the presence of other oligomeric forms. On the opposite, the proteolytically active variant shows the presence of smaller oligomeric forms, e.g. trimers.^52^ Bearing this information in mind, the proteolytically active variant of the HtrA*_Cj_* protein purified under native conditions was tested (Figure 6e). In previous studies, the use of a proteolytically active protein allowed us to observe the clear trimer and hexamer for HtrA of *Stenotrophomonas maltophilia*, despite the fact that inactive variant of protein give us unclear results.^41^ Thus, the wtHtrA*_Cj_* protein, either co-purified with small peptides (substrates), or with additional substrate (β-casein), after digestion of these substrates form a stable hexamer (Figure 6e). Interestingly, we did not observe the presence of 12-mers, which are the active form of the HtrA protein and is visible in the presence of the substrate. This suggests that the substrate was completely digested and stopped allosterically activation of HtrA*_Cj_* to form higher oligomeric forms (Figure 6e). Overall, this gave us information that HtrA*_Cj_* is able to form stable hexamers as well as 12-mers and higher oligomeric forms, and the formation of them can be initiated by a trace amount of small substrates. Interestingly, the first attempts to study HtrA*_Cj_* oligomerization were performed previously using a native gel electrophoresis,^35^ where they observed the formation of 3-, 6- and 12-mers for the proteolytically active variant of protein. However, when they used the proteolytically inactive variant of HtrA*_Cj_* only formation of higher oligomeric forms was observed.^35^ This activation of oligomerization both by full-length substrate and by fragments of peptides was proven for HtrA*_Ec_* (DegP*_Ec_*), it was shown that even small amounts of peptides are able to cause changes in DegP rearrangement.^53^

In conjunction with SEC experiments, cryo-EM of the inactive variant purified in the absence of substrate clearly showed only the dodecameric form of the protein. The use of the proteolytically active form of HtrA*_Cj_* was not possible due to its high tendency to auto-proteolysis. Intriguingly, the dodecamer of HtrA*_Cj_* presents a different organization when compared with the other known structures of DegQ dodecamers, that from *Legionella pneumophila* (PDB ID 4ynn) and DegQ from *E. coli* (PDB ID 4a8a, 4a8b, 4a8c, 4a9g). The major difference between HtrA*_Cj_* and the Legionella dodecamer lies in the different positions of the PDZ2 domains in the trimer, resulting in different inter-trimer contacts. Consequently, the overall external diameter of HtrA*_Cj_* dodecamer is about 16 nm, compared to 14 nm for *Legionella*. The buried surface area following the formation of the dodecamer in HtrA*_Cj_* is significantly smaller, leading to a significantly less stable and more flexible assembly. The dodecamers of *E. coli* DegQ, obtained in the presence of different protein ligands, present a different organization (**Figure S5**). The stability of the assembly has been also tested using molecular dynamics simulation that shows that the dodecamer tends to open, owing to the loose contacts among trimers (**Figure S6**). On the contrary, each trimer appears to be conformationally stable under the dynamics.

We hypothesize that this macromolecular flexibility has a functional meaning, allowing a fast re-assembly and rearrangements from dodecamer to trimers or hexamers, according to the environmental conditions. Therefore, the results of the simulation indicate the ability of the dodecamer to disassemble to a resting state in the absence of a substrate. We are also tempted to speculate that the dodecamer opens, without giving rise to isolated hexamers of trimers, could indicate a path to the formation of higher oligomeric states, for example, with the fusion of two dodecamers.

To complete our knowledge on HtrA*_Cj_*, we also performed a biophysical analysis of HtrA*_Cj_* and measured the melting point of the protein. We compared these data to other bacterial homologs (HtrA*_Hp_*, HtrA*_Ec_*, HtrA*_Sm_*), including their maximum proteolytic activity (Table 1). The melting point for HtrA*_Cj_* was approximately 78°C, and was higher than T_m_ HtrA*_Ec_* and HtrA*_Sm_*, but lower about 10°C than HtrA*_Hp_*.^41,42^ The environmental conditions of *E. coli* and *C. jejuni* are similar, as well as showing maximum proteolytic activity at a temperature of 50–55°C, and similar T*_m_* can be explained by their environmental adaptation. In contrast to *H. pylori*, which lives in extremely adverse stomach conditions and it is necessary that secreted fractions of HtrA*_Hp_* function properly, so Tm over 85°C has great importance in pathogenesis.^42^

Taken together, the present findings have deciphered the biochemical and structural properties of HtrA*_Cj_* and together with the existing knowledge of other bacterial HtrA homologs have enhanced the understanding of the irreplaceable function of this protein as part of the protein quality control system and its important role in pathogenesis of *C. jejuni*.

## Material and methods

### Cloning, expression and purification of HtrAs from C. jejuni, H. pylori and E. coli

The strains and plasmids used in this study are listed in Table 2. The wild-type *htrA* gene of *C. jejuni* (strain 11168) and the protease-inactive *htrA* variant with a single point mutation within the active serine site (S197A) were introduced into the *Nco*I and *Xho*I restriction sites of the pET26b expression plasmid, giving rise to vectors pUZCj4 and pUZCj5, respectively. *E. coli* BL21(DE3)pLysS was transformed with both plasmids and the HtrA proteins with C-terminal 6 x His tags were purified using the pET System (Novagen, San Diego, CA, USA). For this purpose, the bacteria were grown to OD_600 nm_ of 0.8 at 37°C in Luria-Bertani (LB) broth supplemented with kanamycin (50 µg/mL). The steps of purification were carried out exactly as described recently.^34^ Active HtrA and inactive variants (S/A) of *E. coli* (S210A) and *H. pylori* (S221A) were purified as previously described^41,42^ using *E. coli* BL21 (DE3) transformed with the corresponding plasmids (Table 2). All HtrA proteins were purified by nickel-affinity chromatography (Ni-NTA, Qiagen, Germany). The purity of the proteins was at least 95% as estimated by SDS-PAGE. The resulting protein samples were concentrated using Microsep^TM^ Advance Centrifugal Devices 30 K (Pall Corporation, USA).

**Table 2.** Bacterial strains and plasmids

Strain/plasmid	Genotype	Reference/source
*E. coli* BL21DE3	F *^–^ ompT hsdS_B_(r_B_ ^–^ m_B_ ^–^) gal dcm*	Novagen
*E. coli* BL21(DE3)pLysS	F *^–,^ omp*T, *hsd*S_B_(r_B,_ m_B_*^–^*), *dcm, gal*, λ(DE3), pLysS, Cat^R^	Promega
pJS18	pQE60, wt *htrA* from *E. coli* with C- terminal His_6_-tag, Amp^R^	^54^
pJS17	pQE60, *htrA S210*A from *E. coli* with C-terminal His_6_-tag, Amp^R^	^55^
pUZ3	pET26b, *htrAS221A* from the *H. pylori* 26695 strain with C- terminal His_6_-tag, Kan^R^	^42^
pHJS5	pET26b, wt *htrA* from the *H. pylori* 26695 strain with C- terminal His_6_-tag, Kan^R^	^42^
pKB1005	pET28a, S197A *htrA* from the *C. jejuni* NCTC11168 strain, C- terminal 6 x His tag, Kan^R^	^35^
pUZCj4	pET26b, wt *htrA* from the *C. jejuni* NCTC11168 strain, C- terminal 6 x His tag, Kan^R^	^34^
pUZCj5	pET26b, S197A *htrA* from the *C. jejuni* NCTC11168 strain, C- terminal 6 x His tag, Kan^R^	This work
HtrA*_Cj_* S/A GST	pGEX-6P-1, S197A, *htrA* from the *C. jejuni* 81–176 strain, N-term GST-tag, Amp^R^	^29^
HtrA*_Cj_* S/A	pETite N-terminal (Lucigen), S197A *htrA* from *C. jejuni* 81–176 strain, C- terminal 6 x His tag, TEV proteolytic site, Kan^R^	This work

For Cryo-EM experiments, the protease-inactive S197A mutant of HtrA*_Cj_* was used to avoid self–proteolysis. This protein was expressed without the predicted signal peptide (residues 17–472). Prediction of the signal peptide of HtrA*_Cj_* was performed using SignalP 4.0. The gene was introduced into pETite-N-His terminal vector (Lucigen Corporation, USA) with a TEV-protease recognition proteolytic site. The 6His-tag was proteolytically removed after purification. The *E. coli* BL21(DE3) strain was used to overproduce the HtrA*_Cj_* mutant containing N-terminal 6× His tags. For this purpose, the bacteria were grown at 37°C in 2 L LB supplemented with kanamycin (50 µg/mL) to OD of 0.6. HtrA*_Cj_* overexpression was induced by addition of 1 mM isopropyl-β-d-thiogalactopyranoside (IPTG) at 30°C, overnight. Bacteria were harvested at 5000 × *g* for 20 min. The pellet was resuspended in 10 mL of the lysis buffer (20 mM HEPES pH 7.5, 200 mM NaCl) and lysed by sonication. Lysate was cleared by centrifugation 25,000 × *g* for 30 min at 4°C. A nickel-affinity chromatography (Ni–NTA, Qiagen, Germany) under native conditions was used to purify the protein: nonspecifically bound proteins were washed from the loaded resin with washing-buffer (20 mM HEPES pH 7.5, 200 mM NaCl, 20 mM imidazole), and HtrA was eluted by a gradient of elution-buffer (20 mM HEPES pH 7.5, 200 mM NaCl, 20–500 mM imidazole). Protein fractions (checked by SDS-PAGE) were concentrated, changed in buffer with PD-10 column (GE Healthcare Illinois, USA) to eliminate imidazole and incubated with Tev protease overnight at 4°C. To obtain a homogeneous sample, the solution went through a second step of the nickel-affinity column and of a Superdex 200 column (GE Healthcare.

### Analysis of HtrA proteolytic activity

The proteolytic activities of HtrA*_Cj_*, HtrA*_Ec_* and HtrA*_Hp_* were analyzed toward β-casein and native or chemically reduced lysozyme as described.^56,57^ Both wild-type HtrA and inactive S/A mutants were used. The enzymes (0.52 µM) were incubated for 90 min at 37°C with 21 µM β-casein and 35 µM lysozyme in 50 mM HEPES pH 7.4, 200 mM NaCl in a final volume of 200 µL. When indicated, lysozyme was incubated in presence of 7 mM Tris (2-carboxyethyl) phosphine hydrochloride (TCEP). Samples in buffer without HtrA were used as controls. The reactions were terminated by the addition of Laemmli lysis buffer (30 mM Tris- HCl, pH 6.8, 5% glycerol, 1.5% sodium dodecyl sulfate, 0.005% bromophenol blue) and immediate frozen at −20°C. The samples were resolved by 15% SDS-PAGE and the gels were stained with Coomassie Brilliant Blue as described.^41,42^

### Casein zymography

The 50 ng purified active and inactive variant of HtrA proteins from *C. jejuni, E. coli* and *H. pylori* were added to Laemmli buffer. The samples were loaded onto 10% SDS-PAGE gels containing 0.1% casein (Carl Roth, Germany) and then separated under non-reducing conditions. In a next step, in-gel proteins were renatured by incubation of the gel in 2.5% Triton X-100 solution at room temperature for 60 min with gentle agitation and incubated overnight in the developing buffer (50 mM Tris- HCl, pH 7.4, 200 mM NaCl, 5 mM CaCl_2_, 0.02% Brij35) at 37°C.^33,58^ The caseinolytic activity was visualized by staining with 0.5% Coomassie Blue R250 as described.^59^

### Cleavage site-specificity

For cleavage site determination the substrates were treated as above, except for the concentration of enzyme (0.74 µM) and substrate (23 µM β-casein and 73 µM lysozyme) while the NaCl concentration was 100 mM. For reaction with β-casein, samples (200 µL) were withdrawn every 8 min and with lysozyme every 1.5 min. The reactions were stopped by heating the samples for 2 min at 90°C and immediately frozen at −80°C.

The resulting cleavage products were identified by mass spectrometry analysis (LC-MS) at the Laboratory of Mass Spectrometry (IBB PAS, Warsaw) using a nanoAcquity UPLC system (Waters) coupled to an Orbitrap Elite mass spectrometer (Thermo Fisher Scientific). The mass spectrometer was operated in the data-dependent MS2 mode, and data were acquired in the m/z range of 300–2000. Peptides were separated by a 180 min linear gradient of 95% solution A (0.1% v/v formic acid in water) to 35% solution B (acetonitrile and 0.1% formic acid). The measurement of each sample was preceded by three washing runs to avoid cross-contamination. The final MS washing run was searched for the presence of cross-contamination between samples. Data were searched with the MaxQuant (1.6.3.4) search parameters as follows: variable modification: oxidation (M), acetyl (N-term), minimal peptide length 7–25 aa, peptide mass tolerance 20 ppm, fragment mass tolerance 0.5 Da, digestion: unspecific, MS1 resolving power: 70 000, MS2 resolving power 17 500, capillary voltage 3kV, capillary temperature 250 C, collision energy 27%, minimal intensity for detection 10^5^.

### Circular dichroism measurements

Far-UV circular dichroism of the HtrA*_Cj_* S197A protein (0.2 µg/µL in 10 mM Na_2_HPO_4_/NaH_2_PO4, 100 mM NaCl, at pH7.4) was measured between 20°C and 95°C at intervals of 0.5°C and the signals at 218 nm were recorded. The measurement was performed in 1-mm path length cells using a JASCO J-815 (Japan) spectro-polarimeter as described.^60^ The melting point temperature (T_m_) and errors (SD) were calculated from the experimental data as described before.^41,42^

### Cryo-EM image collection

Three µL of HtrA*_Cj_* S197A protein (2 µg/µL) were applied to glow-discharged Quantifoil R2/2 holey carbon grid and vitrified in a Mark IV Vitrobot (FEI). The grids were screened and preliminarily imaged in a Glacios microscope (Thermo Fisher) at 200 keV with a Falcon 2 direct electron camera at 1.2 Å per pixel. 530 movies were collected with 30 frames each and a dose of 1.48 e^−^/Å^2^ per frames. After beam induced motion correction with Motioncor2^61^ and Contrast transfer function (CTF) estimation with Gctf,^62^ 527 micrographs were selected for further particle picking and 2D classification performed in RELION-3.^63^ An initial model generated with EMAN2 was used for 3D classification and refinement.^64^ This dataset leads to a low resolution 8 Å map (data not shown). In order to improve the global resolution, a second dataset under the same grid condition was acquired with a Titan Krios microscope (Thermo Fisher) at 300 KeV with a K2 direct electron camera at 0.827 Å per pixel in CM01 beamline of ESRF. Here, 1,063 movies were collected with 40 frames each and a dose of 1.2 e^−^/Å^2^ per frames. A refinement procedure similar to Glacios data was applied to Krios data set as well. The two datasets were joined following the procedure described^65^ and the 8 Å map previously obtained was used as initial model for the following step. The resulting 3D refinement and post-processing produced a map with a global resolution of 5.8 Å according to the gold standard FSC with 0.143 cutoff (**Table S3 and Fig. S7**).

### Model building and refinement

The initial atomic model for the HtrA*_Cj_* monomer was generated via SwissModel^66^ using the crystal structure of *H. pylori* HtrA (pdb id 5y2d) as a template.^67^ The generated model was rigid body fitted inside the density map using Chimera^68^ and the model was refined by iterative cycles of Phenix real-space refinement^69^ and manual model rebuilding with Coot. Model refinement was carried out using non-crystallographic symmetry and geometry restraints, including secondary structure, rotamer and Ramachandran plot restraints.

### Molecular dynamics simulation

A molecular dynamics simulation of the dodecameric structure was performed with Gromacs 2016.1^70^ using the Amber99 force field^71^ starting from the structure refined in the density map. The model was solvated using the SPC water model in a dodecahedron box with a minimum distance of 1 nm between the protein and the border. Chloride ions were added in order to obtain a zero net charge of the system. After the energy minimization, the temperature was set to 300 K and equilibrated for 100 ps with the Berendsen thermostat.^72^

Similarly, the pressure was equilibrated to 1 atm for 10 ns using the Parrinello-Rahman barostat.^73^ The production simulation was performed for 100 ns, electrostatic and van der Waals interactions were calculated using the Particle Mesh Ewald potential with a 1 nm cutoff.^74^ The mobility of the PDZ domain was determined by calculating the root-mean-square fluctuations (RMSF) of backbone atoms (N, Cα and C atoms) during the molecular dynamics simulations with respect to the reference time-averaged structure.

### Size exclusion chromatography (SEC)

The SEC analysis was performed on Superose 12HR10/30 column (GE Healthcare Life Sciences) equilibrated with 50 mM Na_2_HPO_4_/NaH_2_PO4 pH7.4, 300 mM NaCl as described.^41,42^ The samples (100 µL) containing 41 µM HtrA (obtained by refolded preparation or standard method without refolding step) with or without 41 µM β-casein as a substrate and were analyzed at room temperature at a flow rate 0.3 mL/min using an Agilent High-Performance Liquid Chromatography (HPLC) system. The reaction mixtures were pre-incubated for 10 min at 37°C prior to loading onto the column. The calibration standards were used and contained thyroglobulin (669 kDa), apoferritin (443 kDa), β-amylase (200 kDa), alcohol dehydrogenase (141 kDa), bovine serum (67 kDa), and carbonic anhydrase (29 kDa) (Sigma- Aldrich).

## Supplementary Material

Supplemental Material

## Data Availability

Cryo-EM map and atomic coordinates of HtrA*_Cj_* were deposited at the Electron Microscopy Data Bank (EMDB) with accession codes EMD-11003 and at Protein Data Bank (PDB) with PDB ID: 6Z05
