# Supplementary material for: Functional analysis and cryo-electron microscopy of Campylobacter jejuni serine protease HtrA
Source: Gut Microbes. 2020 Sep 22;12(1):1810532. doi: 10.1080/19490976.2020.1810532 (PMC7524362; doi:10.1080/19490976.2020.1810532)
Supplement: Supplemental Material [file KGMI_A_1810532_SM2524.zip › Supplementary information/Supplementary Materials.docx]

**SUPPLEMENTARY MATERIALS**

**Functional analysis and Cryo-electron microscopy of**

***Campylobacter jejuni* serine protease HtrA**

Urszula Zarzecka, Alessandro Grinzato, Eaazhisai Kandiah, Dominik Cysewski, Paola Berto, Joanna Skorko-Glonek, Giuseppe Zanotti, Steffen Backert

**
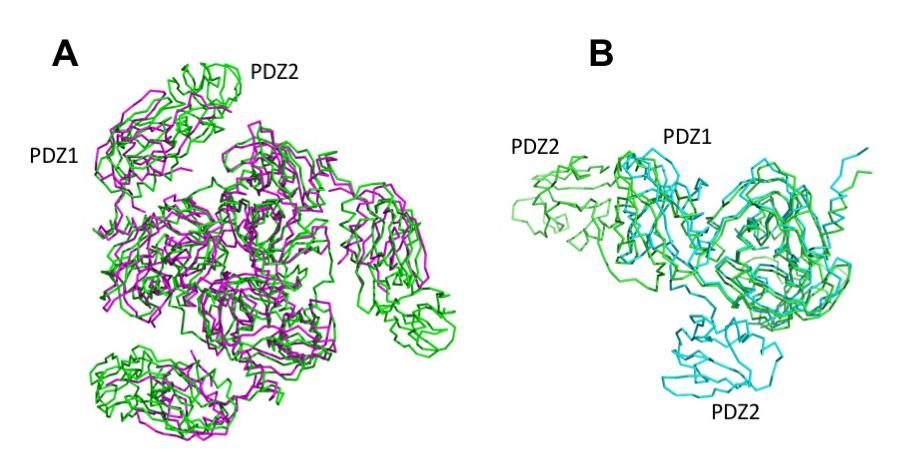
**

**Figure S1.** Cα chain trace of the trimer of HtrA*_Cj_* present in the dodecamer (green) superimposed to (A) a trimer of DegS from *E. coli* (magenta, PDB ID 6ew9) and (B) the monomer of DegP from *E. coli* (cyan, PDB ID 3mh7). In structure 6ew9 the orientation of PDZ1 is similar to that of HtrA*_C_j* and PDZ2 is absent, in 3mh7 PDZ2 is oriented in a fully different way. The X-ray DegQ structure (PDB ID 3sti) from *E. coli* lacks the PDZ1 and PDZ2 domains, and structure 3stj presents only the PDZ1 domain, oriented in a similar way to HtrA*_C_j*.


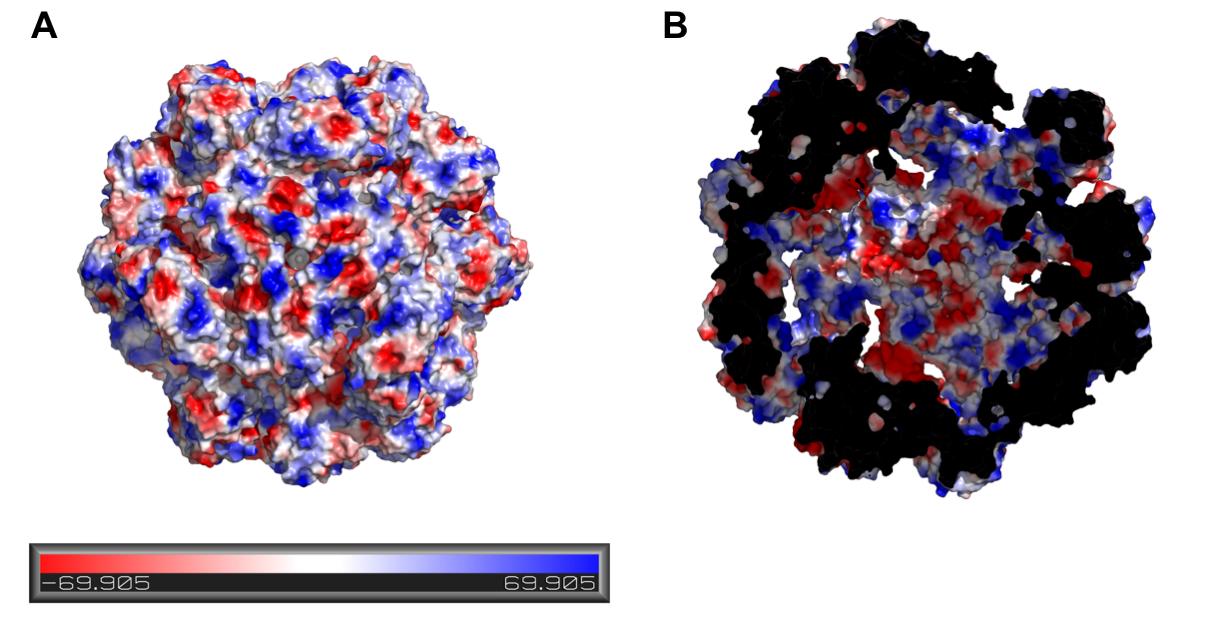


**Figure S2.** **Qualitative electrostatic potential of the dodecameric surface**. Positive and negative charges are in blue and red, respectively. One of the large trimeric openings is shown in the center of (**A**). In panel (**B**), half of the dodecamer is clipped in order to show part of the internal surface.


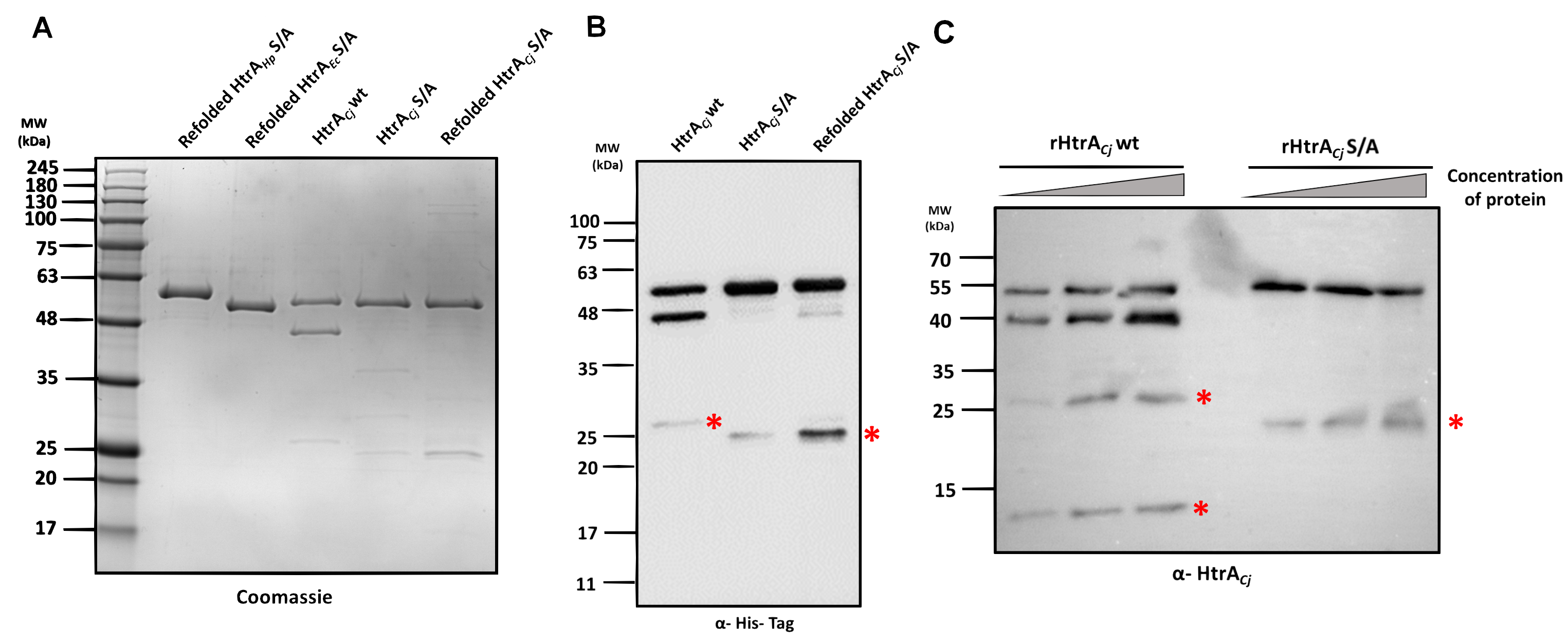


**Figure S3. The profiles of recombinant HtrA*_Cj_* proteins.** The proteins used for the SEC experiments were analyzed by SDS-PAGE (A) and a Western blot was probed with anti-His- Tag antibodies (B) anti-HtrA*_Cj_* antibodies (C). Fragments are visible, and a band of 26 kDa (for active HtrA*_Cj_*) or 24 kDa (for the S/A mutant) was identified as cleavage products of HtrA*_Cj_* (asterisks in panel B and C). The difficulty in separating these shorter fragments was also confirmed by the concentration of the refolded HtrA*_Cj_* S/A sample using filters whose pores should pass a protein less than 30 kDa. However, after this stage, the impurities were still in the HtrA*_Cj_* protein solution.


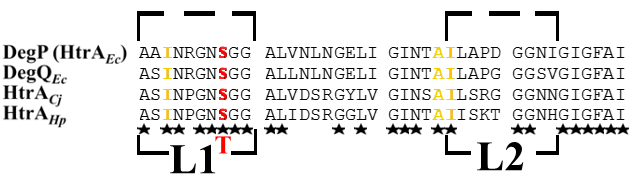


**Figure S4. Comparison of the amino acid sequence of the specificity pocket using selected HtrA homologs: HtrA (DegP) and DegQ *E. coli* (K12 strain), HtrA *C. jejuni* (NCTC11168 strain) and HtrA *H. pylori* (26695 strain).** The regulatory loops (L1, L2) are marked with dashed lines. Asterisks indicate the conserved residues. The S1 specificity pocket is marked in yellow. T- catalytic triad.


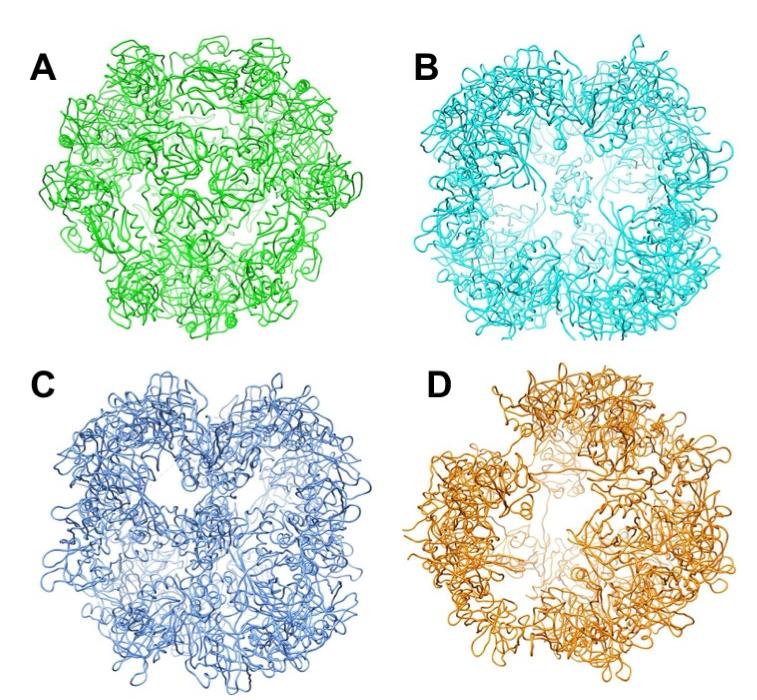


**Figure S5.** Ribbon view of the dodecamer (A) HtrA*_Cj_* , (B) DegQ from *E. coli* in complex with lysozyme (PDB ID 4a8a), (C ) as B, but symmetrized (PDB ID 4a8b) and (D) DegQ from *E. coli* in complex with a binding peptide (PDB ID 4a8c).


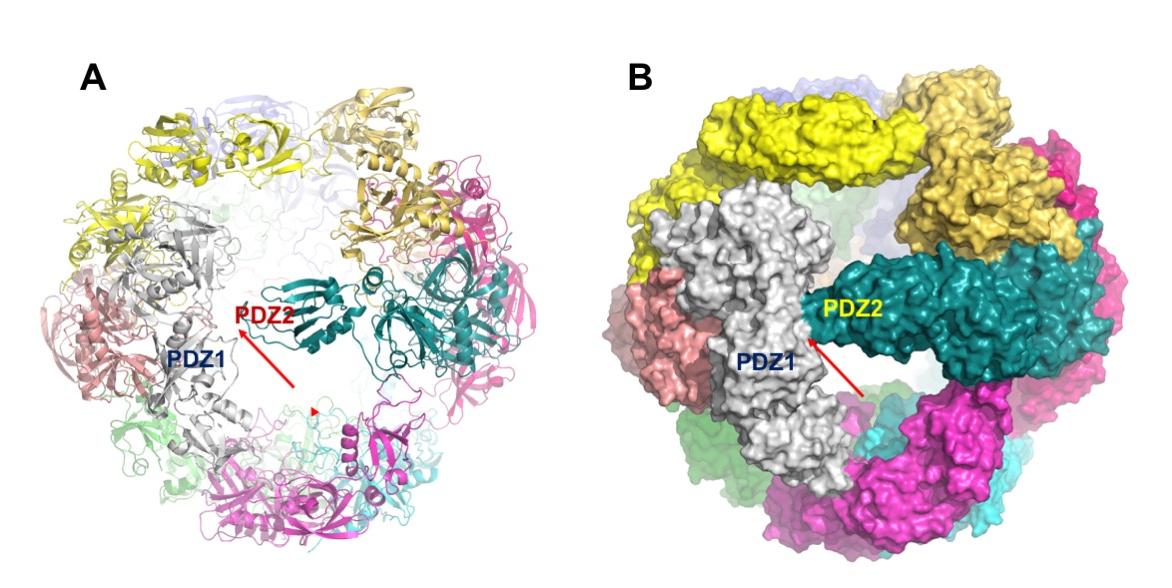


**Figure S6**. **Cartoon view (A) and surface view (B) of the dodecameric assembly of HtrA.** The red arrow indicates the contact area of a PDZ2 domain of one trimer with the PDZ1 of another trimer. These contact areas repeat three times per each trimer.


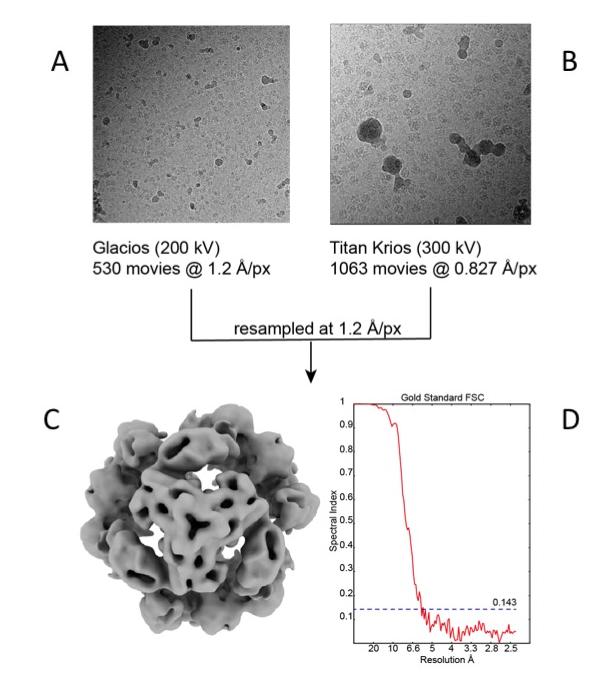


**Fig. S7**. **Data resolution**. (A) end (B) are representative areas from cryo-EM micrographs obtained with the Glacios and Titan Krios microscopes, respectively. An overall view of the dodecamer is shown in (C ), the gold standard Fourier shell correlation curves that indicates a final resolution of 5.8 Å in (D).

**Table S1.** Mass spectrometry data for substrates of HtrA*_Cj_* used to design Figure 2

(provided as separate xls file).

**Table S2.** HtrA*_Cj_* cleavage sites identified in β- casein and lysozyme by mass spectrometry.

| HtrA_Cj_ | | | | | | | | | | | | | | | |
| --- | --- | --- | --- | --- | --- | --- | --- | --- | --- | --- | --- | --- | --- | --- | --- |
| β- casein | | | | | | | | lysozyme | | | | | | | |
| P4 | P3 | P2 | P1 | P1` | P2` | P3` | P4` | P4 | P3 | P2 | P1 | P1` | P2` | P3` | P4` |
| Q | T | P | V | V | V | P | P | F | G | R | C | E | L | A | A |
| V | M | G | V | S | K | V | K | R | C | E | L | A | A | A | M |
| V | S | K | V | K | E | A | M | C | E | L | A | A | A | M | K |
| V | K | E | A | M | A | P | K | E | L | A | A | A | M | K | R |
| P | F | T | E | S | Q | S | L | L | A | A | A | M | K | R | H |
| T | E | S | Q | S | L | T | L | Y | R | G | Y | S | L | G | N |
| L | T | L | T | D | V | E | N | R | G | Y | S | L | G | N | W |
| L | T | D | V | E | N | L | H | G | Y | S | L | G | N | W | V |
| V | E | N | L | H | L | P | L | Y | S | L | G | N | W | V | C |
| P | L | L | Q | S | W | M | H | G | N | W | V | C | A | A | K |
| L | L | Q | S | W | M | H | Q | V | C | A | A | K | F | E | S |
| M | H | Q | P | H | Q | P | L | K | F | E | S | N | F | N | T |
| P | P | T | V | M | F | P | P | N | T | Q | A | T | N | R | N |
| P | Q | S | V | L | S | L | S | T | Q | A | T | N | R | N | T |
| Q | S | V | L | S | L | S | Q | N | R | N | T | D | G | S | T |
| S | V | L | S | L | S | Q | S | I | L | Q | I | N | S | R | W |
| V | L | S | L | S | Q | S | K | N | I | P | C | S | A | L | L |
| L | S | L | S | Q | S | K | V | T | A | S | V | N | C | A | K |
| L | S | Q | S | K | V | L | P | A | S | V | N | C | A | K | K |
| V | L | P | V | P | Q | K | A | S | V | N | C | A | K | K | I |
| P | V | P | Q | K | A | V | P | V | N | C | A | K | K | I | V |
| Y | P | Q | R | D | M | P | I | A | K | K | I | V | S | D | G |
| D | M | P | I | Q | A | F | L | N | A | W | V | A | W | R | N |
| P | I | Q | A | F | L | L | Y | V | A | W | R | N | R | C | K |
| I | Q | A | F | L | L | Y | Q | A | W | R | N | R | C | K | G |
| Q | A | F | L | L | Y | Q | E | W | R | N | R | C | K | G | T |
| A | F | L | L | Y | Q | E | P | R | N | R | C | K | G | T | D |
| Q | E | P | V | L | G | P | V | A | W | I | R | G | C | R | L |
| L | G | P | V | R | G | P | F | W | I | R | G | C | R | L |  |
| P | F | P | I | I | V |  |  |  |  |  |  |  |  |  |  |

**Table S3**. Cryo-EM data and overall statistics for the model (MolProbity)

| **Data collection** |  | |
| --- | --- | --- |
| EM equipment | FEI Glacios | FEI Titian Krios |
| Voltage (kV) | 200 | 300 |
| Detector | Flacon II | K2 |
| Pixel size (Å) | 1.2 | 0.827 |
| # of movie /# of frame | 530 movie /30 frame | 1063 movie /40 frame |
| Electron dose per frame | 1.48 e^−^/Å^2^ | 1.2e^−^/Å^2^ |
| **Reconstruction** |  | |
| Software | RELION 3.0 | |
| Number of particles used | 113843 | |
| Resolution estimates (Å) | Map model FSC (0.143/0.5) 5.9/8.3  Gold Standard FSC (0.143) 5.8 | |
| CC (mask) | 0.76 | |
| Chains | 12 | |
| Total atoms / hydrogens | 39875 /0 | |
| Protin residues | 5208 | |
| Clashscore, all atoms | 38.17 | |
| MolProbity score | 3.43 | |
| Ramachandran plot (%) | Outliers 1.16  Allowed 12.04  Favored 86.81 | |
| Outliers (%) | Rotamer 9.37  Cb 0.00  Peptpide plane 0.00  CaBLAM 8.84 | |
| RMS deviations | Bond lengths 0.005 Å  Angles 1.8° | |

**Supplemental Movie S1: Movie showing the Cryo-EM density map of the dodecamer *C. jejuni* HtrA structure**. The dodecameric assembly of HtrA is presented with each monomer shown with different colors. The density is represented by the white surface.
